# 3-Bromo­anilinium picrate

**DOI:** 10.1107/S1600536809048405

**Published:** 2009-11-25

**Authors:** Yan-jun Li, Bo Zhao

**Affiliations:** a College of Chemical Engineering and Technology, Wuhan University of Science and Technology, Wuhan 430081, People’s Republic of China; b School of Medicine, Wuhan University of Science and Technology, Wuhan 430081, People’s Republic of China

## Abstract

In the title compound, C_6_H_7_BrN^+^·C_6_H_2_N_3_O_7_^−^, the O atoms of two of the nitro groups are disordered over two sites, the ratios of the refined occupancies being 0.72 (6):0.28 (6) and 0.74 (5):0.26 (5). In the crystal structure, the anions and cations are linked *via* inter­molecular N—H⋯O hydrogen bonds into chains along [100]. Further stabilization is provided by weak inter­molecular C—H⋯O hydrogen bonds.

## Related literature

For background information on the crystallization of ammonium salts with picrate derivatives, see: Harrison *et al.* (2007 ▶); Pascard *et al.* (1982 ▶); Pearson *et al.* (2007 ▶); Wang *et al.* (2003 ▶).
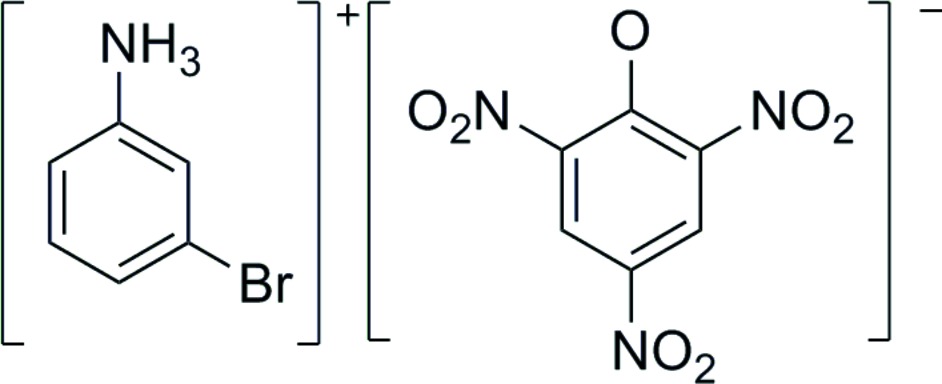


## Experimental

### 

#### Crystal data

C_6_H_7_BrN^+^·C_6_H_2_N_3_O_7_^−^
*M* *_r_* = 401.14
Triclinic, 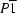

*a* = 4.3515 (3) Å
*b* = 12.0757 (8) Å
*c* = 14.0592 (9) Å
α = 87.783 (1)°
β = 85.945 (1)°
γ = 80.533 (1)°
*V* = 726.61 (8) Å^3^
*Z* = 2
Mo *K*α radiation
μ = 2.88 mm^−1^
*T* = 298 K
0.16 × 0.12 × 0.10 mm

#### Data collection

Bruker SMART APEX CCD diffractometer
Absorption correction: multi-scan (*SADABS*; Sheldrick, 1996 ▶) *T*_min_ = 0.646, *T*_max_ = 0.762
4689 measured reflections
2818 independent reflections
2225 reflections with *I* > 2σ(*I*)
*R*_int_ = 0.090

#### Refinement

*R*[*F* ^2^ > 2σ(*F* ^2^)] = 0.047
*wR*(*F* ^2^) = 0.123
*S* = 0.96
2818 reflections
264 parameters
15 restraints
H atoms treated by a mixture of independent and constrained refinement
Δρ_max_ = 0.69 e Å^−3^
Δρ_min_ = −0.59 e Å^−3^

### 

Data collection: *SMART* (Bruker, 2001 ▶); cell refinement: *SAINT-Plus* (Bruker, 2001 ▶); data reduction: *SAINT-Plus*; program(s) used to solve structure: *SHELXS97* (Sheldrick, 2008 ▶); program(s) used to refine structure: *SHELXL97* (Sheldrick, 2008 ▶); molecular graphics: *PLATON* (Spek, 2009 ▶); software used to prepare material for publication: *PLATON*.

## Supplementary Material

Crystal structure: contains datablocks global, I. DOI: 10.1107/S1600536809048405/lh2949sup1.cif

Structure factors: contains datablocks I. DOI: 10.1107/S1600536809048405/lh2949Isup2.hkl

Additional supplementary materials:  crystallographic information; 3D view; checkCIF report

## Figures and Tables

**Table 1:** Hydrogen-bond geometry (Å, °)

*D*—H⋯*A*	*D*—H	H⋯*A*	*D*⋯*A*	*D*—H⋯*A*
N1—H1*C*⋯O7^i^	0.859 (10)	2.50 (3)	2.966 (12)	115 (3)
N1—H1*C*⋯O1^i^	0.859 (10)	1.934 (14)	2.775 (4)	166 (3)
N1—H1*A*⋯O1	0.856 (10)	1.958 (17)	2.766 (4)	157 (3)
N1—H1*B*⋯O6^ii^	0.862 (10)	2.28 (2)	3.047 (12)	148 (3)
C11—H11⋯O5^iii^	0.93	2.45	3.296 (4)	152
C4—H4⋯O3^iv^	0.93	2.57	3.273 (7)	133

Symmetry codes: (i) 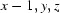; (ii) 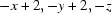; (iii) 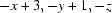; (iv) 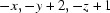.

**Fig. 1.**
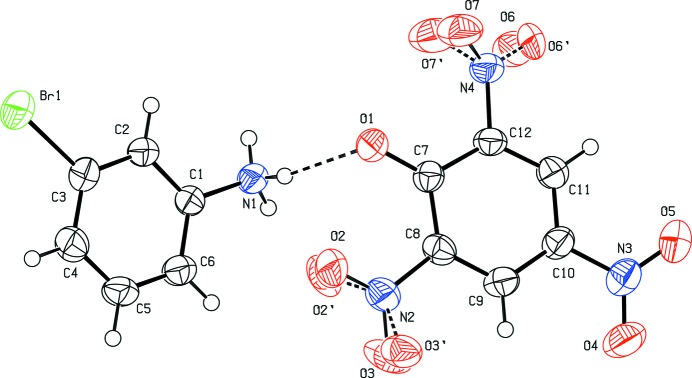
The asymmetric unit with the atom-numbering scheme. The displacement ellipsoids are drawn at the 30% probability level. A hydrogen bond is shown by a dashed line. The minor components of disorder are indicated by primed atom labels.

**Fig. 2.**
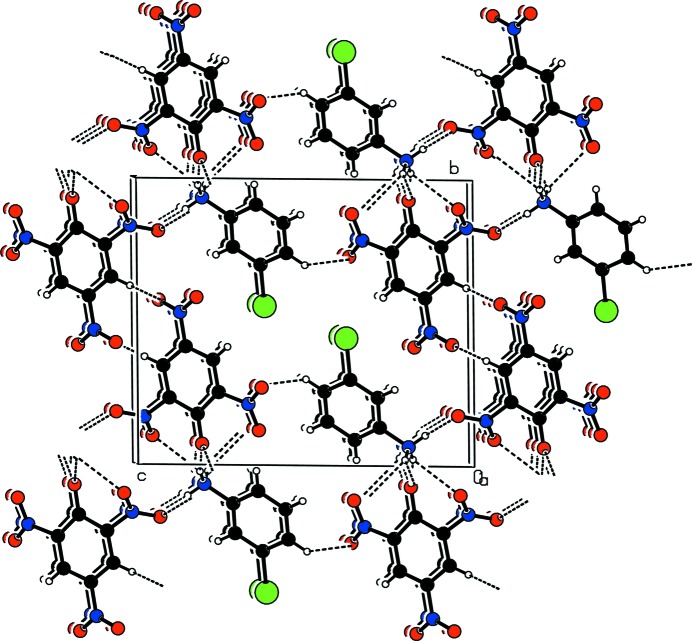
Part of the crystal structure with hydrogen bonds shown as dashed lines.

## supplementary crystallographic information

### Comment

The interaction of picric acid and amines has been widely studied and salt formation takes place readily with very low activation energy. Ammonium salts are easy to crystallize and purify when picrate derivatives are present (Pascard *et al.*,1982;Wang *et al.*,2003; Pearson *et al.*, 2007; Harrison *et al.*, 2007). Herein, we report the crystal structure of the title compound.

In the title compound, the proton has been transferred from the phenolic hydroxylic group to the amine group, resulting in an 1:1 organic salt (Fig.1). In the crystal structure, the molecular components are linked together by intermolecular N—H···O hydrogen bonds forming a one-dimensional chain running parallel to [100]. Adjacent chains are further linked by two weak intermolecular C—H···O hydrogen bonds (see Fig. 2).

### Experimental

Picric acid (0.6873 g, 3 mmol) and 3-Bromoaniline (0.5161 g, 3 mmol) were mixed in 10 ml ethanol. The mixture was kept at room temperature for ten days. Yellow block-shaped crystals suitable for the single-crystal X-ray diffraction were collected from the bottom of the vessel.

### Refinement

In the picrate anion two of the nitro groups oxygen atoms are disordered over two positions with refined occupancies 0.72 (6):0.28 (6) and 0.74 (5):0.26 (5) for O2/O3:O2'/O3' and O6/O7:O6'/O7', respectively.

The carbon-bound hydrogen atoms were placed in ideal positions with C—H=0.93Å and *U*_iso_(H) = 1.2*U*_eq_(C). H1A, H1B and H1C atoms were located in a difference map and refined with the restraint of N—H = 0.86 (1)Å and *U*_iso_(H) = 1.2*U*_eq_(N).

### Crystal data

**Table:**

C_6_H_7_BrN^+^·C_6_H_2_N_3_O_7_^−^	*Z* = 2
*M**_r_* = 401.14	*F*(000) = 400
Triclinic, *P*1	*D*_x_ = 1.833 Mg m^−^^3^
Hall symbol: -P 1	Mo *K*α radiation, λ = 0.71073 Å
*a* = 4.3515 (3) Å	Cell parameters from 2179 reflections
*b* = 12.0757 (8) Å	θ = 2.3–27.6°
*c* = 14.0592 (9) Å	µ = 2.88 mm^−^^1^
α = 87.783 (1)°	*T* = 298 K
β = 85.945 (1)°	Block, yellow
γ = 80.533 (1)°	0.16 × 0.12 × 0.10 mm
*V* = 726.61 (8) Å^3^	

### Data collection

**Table:**

Bruker SMART APEX CCD diffractometer	2818 independent reflections
Radiation source: fine focus sealed Siemens Mo tube	2225 reflections with *I* > 2σ(*I*)
graphite	*R*_int_ = 0.090
0.3° wide ω exposures scans	θ_max_ = 26.0°, θ_min_ = 2.2°
Absorption correction: multi-scan (*SADABS*; Sheldrick, 1996)	*h* = −5→5
*T*_min_ = 0.646, *T*_max_ = 0.762	*k* = −14→12
4689 measured reflections	*l* = −17→16

### Refinement

**Table:**

Refinement on *F*^2^	Primary atom site location: structure-invariant direct methods
Least-squares matrix: full	Secondary atom site location: difference Fourier map
*R*[*F*^2^ > 2σ(*F*^2^)] = 0.047	Hydrogen site location: inferred from neighbouring sites
*wR*(*F*^2^) = 0.123	H atoms treated by a mixture of independent and constrained refinement
*S* = 0.96	*w* = 1/[σ^2^(*F*_o_^2^) + (0.0686*P*)^2^] where *P* = (*F*_o_^2^ + 2*F*_c_^2^)/3
2818 reflections	(Δ/σ)_max_ < 0.001
264 parameters	Δρ_max_ = 0.69 e Å^−^^3^
15 restraints	Δρ_min_ = −0.59 e Å^−^^3^

### Special details

**Table:**

Geometry. All e.s.d.'s (except the e.s.d. in the dihedral angle between two l.s. planes) are estimated using the full covariance matrix. The cell e.s.d.'s are taken into account individually in the estimation of e.s.d.'s in distances, angles and torsion angles; correlations between e.s.d.'s in cell parameters are only used when they are defined by crystal symmetry. An approximate (isotropic) treatment of cell e.s.d.'s is used for estimating e.s.d.'s involving l.s. planes.
Refinement. Refinement of *F*^2^ against ALL reflections. The weighted *R*-factor *wR* and goodness of fit *S* are based on *F*^2^, conventional *R*-factors *R* are based on *F*, with *F* set to zero for negative *F*^2^. The threshold expression of *F*^2^ > σ(*F*^2^) is used only for calculating *R*-factors(gt) *etc*. and is not relevant to the choice of reflections for refinement. *R*-factors based on *F*^2^ are statistically about twice as large as those based on *F*, and *R*- factors based on ALL data will be even larger.

### Fractional atomic coordinates and isotropic or equivalent isotropic displacement parameters (Å^2^)

**Table:**

	*x*	*y*	*z*	*U*_iso_*/*U*_eq_	Occ. (<1)
Br1	0.31978 (9)	1.44651 (3)	0.38403 (3)	0.0620 (2)	
C1	0.2439 (7)	1.1358 (3)	0.2852 (2)	0.0365 (6)	
C2	0.3254 (7)	1.2405 (3)	0.2916 (2)	0.0400 (7)	
H2	0.4488	1.2694	0.2432	0.048*	
C3	0.2197 (7)	1.3015 (3)	0.3716 (2)	0.0418 (7)	
C4	0.0376 (8)	1.2589 (3)	0.4453 (2)	0.0488 (8)	
H4	−0.0312	1.3006	0.4992	0.059*	
C5	−0.0383 (8)	1.1533 (3)	0.4364 (2)	0.0500 (8)	
H5	−0.1593	1.1236	0.4851	0.060*	
C6	0.0623 (8)	1.0912 (3)	0.3568 (2)	0.0451 (7)	
H6	0.0090	1.0204	0.3512	0.054*	
C7	0.9423 (7)	0.8220 (3)	0.1767 (2)	0.0376 (7)	
C8	0.7948 (7)	0.7451 (3)	0.2374 (2)	0.0401 (7)	
C9	0.8229 (8)	0.6320 (3)	0.2218 (2)	0.0432 (7)	
H9	0.7200	0.5862	0.2632	0.052*	
C10	1.0069 (8)	0.5873 (3)	0.1436 (2)	0.0434 (7)	
C11	1.1497 (7)	0.6552 (3)	0.0790 (2)	0.0433 (7)	
H11	1.2660	0.6253	0.0253	0.052*	
C12	1.1162 (7)	0.7672 (3)	0.0960 (2)	0.0398 (7)	
N1	0.3525 (7)	1.0693 (2)	0.20133 (19)	0.0421 (6)	
H1A	0.520 (5)	1.025 (3)	0.215 (2)	0.051*	
H1B	0.400 (8)	1.110 (3)	0.1527 (17)	0.051*	
H1C	0.229 (7)	1.025 (2)	0.187 (2)	0.051*	
N2	0.6000 (7)	0.7845 (3)	0.32217 (19)	0.0484 (7)	
N3	1.0392 (7)	0.4679 (3)	0.1282 (2)	0.0503 (7)	
N4	1.2620 (7)	0.8348 (2)	0.02367 (18)	0.0490 (7)	
O1	0.9193 (5)	0.9265 (2)	0.18883 (16)	0.0489 (6)	
O2	0.623 (5)	0.8764 (8)	0.3531 (10)	0.066 (3)	0.72 (6)
O3	0.419 (6)	0.7259 (14)	0.3589 (15)	0.081 (4)	0.72 (6)
O2'	0.506 (13)	0.8824 (11)	0.334 (2)	0.074 (8)	0.28 (6)
O3'	0.555 (13)	0.7092 (12)	0.3811 (14)	0.058 (8)	0.28 (6)
O4	0.8643 (8)	0.4144 (2)	0.1743 (2)	0.0706 (8)	
O5	1.2388 (7)	0.4267 (2)	0.0683 (2)	0.0661 (7)	
O6	1.223 (5)	0.8179 (12)	−0.0605 (4)	0.068 (3)	0.74 (5)
O7	1.419 (3)	0.9014 (12)	0.0496 (6)	0.064 (3)	0.74 (5)
O6'	1.340 (11)	0.798 (3)	−0.0553 (15)	0.073 (8)	0.26 (5)
O7'	1.27 (2)	0.935 (3)	0.039 (3)	0.099 (14)	0.26 (5)

### Atomic displacement parameters (Å^2^)

**Table:**

	*U*^11^	*U*^22^	*U*^33^	*U*^12^	*U*^13^	*U*^23^
Br1	0.0788 (3)	0.0419 (2)	0.0677 (3)	−0.01847 (19)	0.0063 (2)	−0.01619 (18)
C1	0.0392 (15)	0.0353 (16)	0.0344 (15)	−0.0054 (13)	0.0003 (12)	−0.0009 (12)
C2	0.0447 (16)	0.0371 (17)	0.0382 (15)	−0.0100 (14)	0.0045 (13)	0.0000 (13)
C3	0.0465 (17)	0.0359 (16)	0.0429 (16)	−0.0051 (14)	−0.0051 (13)	−0.0030 (13)
C4	0.059 (2)	0.050 (2)	0.0356 (16)	−0.0058 (16)	0.0021 (14)	−0.0039 (14)
C5	0.062 (2)	0.050 (2)	0.0379 (17)	−0.0161 (17)	0.0096 (15)	0.0034 (15)
C6	0.0538 (18)	0.0408 (18)	0.0416 (17)	−0.0132 (15)	0.0024 (14)	0.0018 (14)
C7	0.0412 (15)	0.0368 (17)	0.0363 (15)	−0.0098 (13)	−0.0032 (12)	−0.0045 (12)
C8	0.0424 (16)	0.0492 (19)	0.0296 (14)	−0.0107 (14)	0.0006 (12)	−0.0048 (13)
C9	0.0506 (18)	0.0424 (18)	0.0389 (16)	−0.0159 (15)	−0.0021 (13)	0.0025 (14)
C10	0.0568 (19)	0.0354 (17)	0.0389 (16)	−0.0107 (15)	−0.0024 (14)	−0.0016 (13)
C11	0.0495 (18)	0.0429 (18)	0.0375 (16)	−0.0091 (15)	0.0028 (13)	−0.0048 (14)
C12	0.0448 (16)	0.0412 (18)	0.0347 (15)	−0.0139 (14)	0.0033 (12)	0.0007 (13)
N1	0.0517 (16)	0.0350 (15)	0.0405 (14)	−0.0121 (12)	0.0048 (12)	−0.0042 (11)
N2	0.0560 (17)	0.0534 (19)	0.0354 (14)	−0.0103 (15)	0.0051 (12)	−0.0044 (14)
N3	0.0647 (18)	0.0412 (16)	0.0463 (15)	−0.0100 (14)	−0.0090 (14)	−0.0012 (13)
N4	0.0648 (18)	0.0423 (17)	0.0408 (16)	−0.0171 (14)	0.0113 (13)	−0.0026 (12)
O1	0.0499 (13)	0.0405 (13)	0.0580 (14)	−0.0141 (10)	0.0069 (10)	−0.0112 (11)
O2	0.092 (7)	0.048 (4)	0.053 (4)	−0.007 (3)	0.021 (4)	−0.012 (2)
O3	0.078 (8)	0.106 (5)	0.067 (6)	−0.051 (5)	0.035 (6)	−0.029 (4)
O2'	0.079 (18)	0.069 (10)	0.054 (11)	0.028 (8)	0.028 (10)	0.016 (7)
O3'	0.071 (16)	0.066 (8)	0.043 (7)	−0.032 (8)	0.010 (8)	−0.012 (5)
O4	0.099 (2)	0.0506 (16)	0.0676 (17)	−0.0332 (16)	0.0046 (15)	0.0009 (13)
O5	0.0760 (18)	0.0444 (15)	0.0747 (17)	−0.0016 (13)	0.0045 (14)	−0.0115 (13)
O6	0.106 (8)	0.061 (5)	0.040 (3)	−0.029 (5)	0.015 (3)	−0.006 (2)
O7	0.067 (4)	0.071 (5)	0.064 (3)	−0.039 (4)	0.003 (3)	−0.002 (3)
O6'	0.097 (18)	0.044 (9)	0.068 (11)	−0.001 (11)	0.047 (9)	−0.013 (7)
O7'	0.14 (4)	0.084 (13)	0.085 (14)	−0.067 (17)	0.039 (19)	−0.019 (12)

### Geometric parameters (Å, °)

**Table:**

Br1—C3	1.890 (3)	C10—C11	1.383 (4)
C1—C2	1.376 (4)	C10—N3	1.449 (4)
C1—C6	1.382 (4)	C11—C12	1.365 (5)
C1—N1	1.459 (4)	C11—H11	0.9300
C2—C3	1.377 (4)	C12—N4	1.455 (4)
C2—H2	0.9300	N1—H1A	0.856 (10)
C3—C4	1.394 (4)	N1—H1B	0.862 (10)
C4—C5	1.382 (5)	N1—H1C	0.859 (10)
C4—H4	0.9300	N2—O2'	1.200 (10)
C5—C6	1.377 (5)	N2—O3	1.218 (5)
C5—H5	0.9300	N2—O2	1.229 (6)
C6—H6	0.9300	N2—O3'	1.237 (9)
C7—O1	1.266 (4)	N3—O4	1.215 (4)
C7—C8	1.435 (4)	N3—O5	1.223 (4)
C7—C12	1.439 (4)	N4—O6'	1.218 (10)
C8—C9	1.375 (5)	N4—O7	1.220 (6)
C8—N2	1.461 (4)	N4—O6	1.236 (6)
C9—C10	1.386 (4)	N4—O7'	1.240 (10)
C9—H9	0.9300		
			
C2—C1—C6	121.6 (3)	C12—C11—H11	120.7
C2—C1—N1	119.5 (2)	C10—C11—H11	120.7
C6—C1—N1	118.8 (3)	C11—C12—C7	125.2 (3)
C1—C2—C3	118.4 (3)	C11—C12—N4	115.8 (3)
C1—C2—H2	120.8	C7—C12—N4	118.9 (3)
C3—C2—H2	120.8	C1—N1—H1A	108 (3)
C2—C3—C4	121.5 (3)	C1—N1—H1B	112 (2)
C2—C3—Br1	120.4 (2)	H1A—N1—H1B	107 (3)
C4—C3—Br1	118.1 (2)	C1—N1—H1C	114 (2)
C5—C4—C3	118.5 (3)	H1A—N1—H1C	104 (4)
C5—C4—H4	120.8	H1B—N1—H1C	111 (3)
C3—C4—H4	120.8	O2'—N2—O3	111.6 (16)
C6—C5—C4	121.0 (3)	O3—N2—O2	122.5 (6)
C6—C5—H5	119.5	O2'—N2—O3'	123.8 (14)
C4—C5—H5	119.5	O2—N2—O3'	117.4 (10)
C5—C6—C1	119.0 (3)	O2'—N2—C8	121.9 (12)
C5—C6—H6	120.5	O3—N2—C8	119.0 (4)
C1—C6—H6	120.5	O2—N2—C8	118.5 (5)
O1—C7—C8	125.7 (3)	O3'—N2—C8	114.2 (12)
O1—C7—C12	122.5 (3)	O4—N3—O5	123.5 (3)
C8—C7—C12	111.8 (3)	O4—N3—C10	118.4 (3)
C9—C8—C7	124.2 (3)	O5—N3—C10	118.1 (3)
C9—C8—N2	115.3 (3)	O6'—N4—O7	114.7 (18)
C7—C8—N2	120.5 (3)	O7—N4—O6	124.7 (6)
C8—C9—C10	119.1 (3)	O6'—N4—O7'	119.8 (15)
C8—C9—H9	120.5	O6—N4—O7'	113 (2)
C10—C9—H9	120.5	O6'—N4—C12	120.1 (15)
C11—C10—C9	121.1 (3)	O7—N4—C12	118.3 (4)
C11—C10—N3	120.0 (3)	O6—N4—C12	117.0 (6)
C9—C10—N3	118.9 (3)	O7'—N4—C12	119.6 (11)
C12—C11—C10	118.5 (3)	C7—O1—H1A	123.4 (12)

### Hydrogen-bond geometry (Å, °)

**Table:**

*D*—H···*A*	*D*—H	H···*A*	*D*···*A*	*D*—H···*A*
N1—H1C···O7^i^	0.86 (1)	2.50 (3)	2.966 (12)	115 (3)
N1—H1C···O1^i^	0.86 (1)	1.93 (1)	2.775 (4)	166 (3)
N1—H1A···O1	0.86 (1)	1.96 (2)	2.766 (4)	157 (3)
N1—H1B···O6^ii^	0.86 (1)	2.28 (2)	3.047 (12)	148 (3)
C11—H11···O5^iii^	0.93	2.45	3.296 (4)	152
C4—H4···O3^iv^	0.93	2.57	3.273 (7)	133

Symmetry codes: (i) *x*−1, *y*, *z*; (ii) −*x*+2, −*y*+2, −*z*; (iii) −*x*+3, −*y*+1, −*z*; (iv) −*x*, −*y*+2, −*z*+1.
